# Duodenal microbiota composition and mucosal homeostasis in pediatric celiac disease

**DOI:** 10.1186/1471-230X-13-113

**Published:** 2013-07-11

**Authors:** Jing Cheng, Marko Kalliomäki, Hans GHJ Heilig, Airi Palva, Hannu Lähteenoja, Willem M de Vos, Jarkko Salojärvi, Reetta Satokari

**Affiliations:** 1 Department of Veterinary Biosciences, University of Helsinki, P.O. Box 66, Helsinki FI-00014, Finland; 2 Department of Pediatrics, University of Turku and Turku University Central Hospital, P.O. Box 52, Turku 20521, Finland; 3 Functional Foods Forum, 20014 University of Turku, Turku, Finland; 4 Laboratory of Microbiology, Wageningen University, Dreijenplein 10, Wageningen 6703 HB, The Netherlands; 5 Department of Internal Medicine, Turku University Central Hospital, P.O. Box 52, Turku 20521, Finland; 6 Haartman Institute, University of Helsinki, P.O. Box 21, Helsinki FI-00014, Finland

**Keywords:** Celiac disease, Microbiota, Gene expression, Duodenum, Host-microbe cross-talk

## Abstract

**Background:**

Celiac disease (CD) is an autoimmune disorder of the small intestine which is triggered by dietary gluten in genetically predisposed (HLA-DQ2/DQ8 positive) individuals. Only a fraction of HLA-DQ2/DQ8 positive individuals develop CD indicating that other factors have a role in the disorder. Several studies have addressed intestinal microbiota aberrancies in pediatric CD, but the results are inconsistent. Previously, we demonstrated that pediatric CD patients have lower duodenal expression of TLR2 and higher expression of TLR9 as compared to healthy controls (HC) indicating that microbiota may have a role in CD.

**Methods:**

We used bacterial phylogenetic microarray to comprehensively profile the microbiota in duodenal biopsies of CD (n = 10) and HC (n = 9) children. The expression of selected mucosa-associated genes was assessed by qRT-PCR in CD and HC children and in treated CD adults (T-CD, n = 6) on gluten free diet.

**Results:**

The overall composition, diversity and the estimated microbe associated molecular pattern (MAMP) content of microbiota were comparable between CD and HC, but a sub-population profile comprising eight genus-like bacterial groups was found to differ significantly between HC and CD. In HC, increased TLR2 expression was positively correlated with the expression of tight junction protein ZO-1. In CD and T-CD, the expression of IL-10, IFN-g and CXCR6 were higher as co5mpared to HC.

**Conclusions:**

The results suggest that microbiota and altered expression of mucosal receptors have a role in CD. In CD subjects, the increased expression of IL-10 and IFN-g may have partly resulted from the increased TLR9 expression and signaling.

## Background

Celiac disease (CD) is a common chronic immune-mediated, inflammatory disorder of the small intestine induced by intolerance to gluten-containing dietary products [1,2]. When a CD patient consumes gluten, an inflammatory cascade occurs in the small intestinal mucosa, eventually resulting in an active disease that is characterized by villous atrophy, crypt hyperplasia and increased numbers of lymphocytes in the lamina propria [1,3]. Untreated CD is manifested by gastrointestinal symptoms, malabsorption and even malnutrition [1,3] and also by extra-intestinal symptoms such as dermititis herpetiformis (skin rash) in some individuals [3]. Both genetic predisposition and environmental factors are considered to be involved in the development of CD [4]. Individuals who carry the alleles human leukocyte antigen (HLA)-DQ2 or HLA-DQ8 have an increased risk of developing the disease, but only less than one tenth of them eventually get CD, indicating that other genetic factors and/or environmental factors are also important in the pathogenesis [5]. Recently, 13 new CD risk loci were identified, bringing the number of known CD loci to 40 and giving a refined picture of the genetic risk of CD [6]. Most of these loci contain candidate genes of immunological function, but the pathways leading from genetic predisposition to an affected person are poorly understood [6,7]. The intestinal microbiota is a major dictator of the antigen milieu of enterocytes, and it may have a role in the CD pathogenesis.

Gut colonization starts immediately after birth, and considerable microbiota maturation takes place during the first years of life followed by a gradual microbiota succession until young adulthood [8-10]. A balanced commensal microbiota contributes to the physiological development of the gut and the maturation of the immune system; thereby, alterations in the intestinal microbiota could play a role in the onset of different diseases, including CD [3,11]. Increased bacterial diversity and changes in several bacterial groups in the microbiota of pediatric CD patients have been reported in several studies [12-16]. However, other recent studies have failed to show major microbiota differences between children with and without CD [17-19]. Two recent studies have addressed the microbiota in infants with a genetic predisposition to CD [4,11]. Both of these reported the microbiota of predisposed infants to be different from that of non-predisposed, but while Sellitto et al. reported a reduction or lack of *Bacteroides* in predisposed infants [4], De Palma et al. found that *Bacteroides fragilis* and staphylococci were increased and bifidobacteria were reduced in genetically susceptible infants [11]. Although the idea that the microbiota is involved in the etiology of CD has been addressed in numerous studies, the results on specific CD-associated microbiota changes remain inconclusive.

Recently, we demonstrated that pediatric CD patients have decreased duodenal expression of Toll-like receptor 2 (TLR2) and the negative regulator of Toll-receptor signaling (Tollip), and increased expression of TLR9 and interleukin 8 (IL-8), which is a marker of intestinal inflammation [18]. TLRs are a family of pattern recognition receptors, which recognize conserved microbe-associated motifs such as lipopolysaccharides (LPS, ligand for TLR4), lipoproteins and lipoteichoeic acids (LTA, ligand for TLR2), flagellin (TLR5) and nucleic acid motifs (TLR3, 7 and 9). Intestinal epithelial homeostasis is dependent on the activation of TLRs at adequate levels in order to keep defense against microbes balanced and to avoid an excessive inflammatory response to gut commensals [20,21]. Further, TLR signaling is known to affect epithelial barrier function by having a bearing on the expression of tight junction proteins, mucus protein mucin 2 and antimicrobial peptides such as RegIII-γ [20,21]. Thus, our previous results suggested that microbiota may play a role in the etiology of CD, but we were unable to reveal aberrancies in the duodenal mucosal microbiota of pediatric CD by targeting selected bacterial groups by quantitative PCR (qPCR) [18].

The objectives of the present study were to comprehensively characterize the total duodenal mucosal microbiota and to re-evaluate the possible microbiota differences in pediatric CD patients and healthy controls by using a high-throughput bacterial phylogenetic microarray (HITChip). Further, the expression of nine mucosa-associated genes, IL-10, interferon-gamma (IFN-γ), tumor necrosis factor alpha (TNF-α), a tight junction protein zonula occludens-1 (ZO-1), a gap junction protein connexin-43 (Cx43), a mucus protein mucin 2 (MUC2), an antimicrobial peptide RegIIIγ, a chemokine CXCL16 and its receptor CXCR6 were measured by using a quantitative reverse transcription-PCR (qRT-PCR).

## Methods

### Study subjects and DNA and RNA extraction

Duodenal biopsy samples were collected from 20 Finnish children and 6 Finnish adults: 10 children with newly diagnosed CD (median age 9.5 ± 4.1 y; 3-14 y, 4 males and 6 females) before the implementation of gluten-free diet (GFD), 10 healthy control (HC) children (median age 8.5 ± 3.8 y; 4-16y, 4 males and 6 females) with gastrointestinal complaints or other reasons for esophagogastroduodenoscopy (abdominal pain - 4 patients, gastroesophageal reflux disease – 2 patients, growth retardation – 2 patients esophagitis -1 patient, achalasia – 1 patient) and 6 adults (median age 46 ± 11.4 y; 30-60 y) with CD who had been on a GFD at least for a year (treated CD, T-CD). All HC and T-CD subjects had both negative celiac serology and normal small intestinal mucosa (Marsh 0 lesions), while CD patients had both positive celiac serology markers (anti-tissue transglutaminase antibodies and/or anti-endomysium antibodies) and villous atrophy and crypt hyperplasia (Marsh III lesions) in duodenal biopsy. The study subjects were the same as in our previous study [18] and the same DNA and RNA preparations were used in this study. The DNA and RNA extraction and purification protocols were described in detail earlier [18].

### Quantitative reverse-transcriptase-PCR (qRT-PCR)

Gene expression assays were performed using comparative Ct (threshold cycle)-method with ABI 7300 Real Time PCR System. (Applied Biosystems/Life Technologies Corporation, Carlsbad, CA). Taqman Gene Expression Assays (Applied Biosystems) used in analyses were: IL-10, assay ID: Hs00174086_m1; IFN-g, assay ID: Hs00174143_m1; TNF-α assay ID: Hs00174128; ZO-1, assay ID: Hs01551861_m1; Cx43, assay ID: Hs00748445_s1; MUC2, assay ID: Hs03005103_g1; RegIIIγ, assay ID: Hs01595405_m1; CXCL16, assay ID: Hs00222859_m1; CXCR6, assay ID: Hs01890898_s1. Gene expression assays were performed according to kit’s protocol. Reactions were run in three replicates in a total volume of 20 μl or 50 μl with 25 ng of cDNA in each. Thermal cycler conditions used were 1) 50°C for 2 min, 2) 95°C for 10 min, 3) 95°C for 15 sec, 4) 60°C for 1 min. Steps 3 and 4 were repeated 40 times. Gene expression of 18S rRNA was used as an endogenous control (a house-keeping gene) to normalize the gene expression. Negative control and Universe Human Reference RNA (Agilent Technologies, Santa Clara, CA) as a control RNA were included in every PCR run. Results were analyzed with RQ-Study program (Applied Biosystems) to receive Ct values for all samples. The relative expression of genes was then calculated as described in detail previously [18].

### Microbiota analysis by phylogenetic microarray

The microbiota was analyzed with the bacterial phylogenetic microarray –the HITChip (Human Intestinal Tract Chip) [22-24]. This microarray consists of over 4 800 oligonucleotide probes targeting the V1 and V6 hypervariable regions of the 16S rRNA gene of 1038 intestinal phylotypes [22-24]. It allows a comprehensive and high-resolution analysis of the microbiota composition at different taxonomic levels. Genus-like level (L2) taxa correspond to bacteria having 90% or higher similarity in their 16S rRNA gene, whereas L1 taxa correspond to a phylum-like level [22].

The HITChip measurements were performed as previously described [22-24]. In brief, amplification of 16S rRNA gene was carried out from 100-200 ng of DNA with primer T7 prom-Bact-27 F and Bact-1369R [25], followed by *in vitro* transcription, dye labeling, fragmentation, and hybridization, as described earlier [22]. The arrays were scanned with Agilent DNA Microarray Scanner G2505C (Agilent, USA) and the intensity values for each image were extracted from the generated images using Agilent Extraction Software, version 10.7.3.1. Normalization and quality control of HITChip array data were performed with scripts in R statistical software, as described earlier [22,24,26]. The technical replicates having a Pearson correlation over 0.94 were selected for further analysis and the replicates were averaged. The between sample normalization was carried out with min-max algorithm [27,28]. Signal intensity threshold was applied to reduce experimental and possible cross-hybridization noise, as described previously [22].

### Estimation of the microbe associated molecular pattern (MAMP) content

The relative content of selected MAMPs was estimated from the HITChip profiles summarizing the abundance of Gram-positive, Gram–negative or flagellated genus-like bacterial groups. The abundance of Gram-positive bacteria is assumed to reflect the load of LTA, i.e. TLR2 ligands, the abundance of Gram-negative bacteria the load of LPS i.e. TLR4 ligands, and the abundance of flagellated bacteria the load of TLR5 ligands. The general GC% of the microbiome was estimated based on the genomic GC-content of representative species from each of the genus-like group (Additional file 1: Table S1), weighted by the relative abundance of each of these groups in the total microbiota. The GC% is taken to reflect the load of unmethylated CpG motifs, i.e. TLR9 ligands, as a high GC content of a bacterial genome correlates with a higher number of CpG motifs in the genome (Kant R, de Vos WM, Palva A, Satokari R, unpublished results) [29,30].

### Statistical analysis

The data analysis was performed in R version 2.15.1 (R Development CT 2012). The sum of signal intensities for probes targeting a genus-like group was used as a quantitative measure of the abundance of the group in a sample [24]. When computing the signal at higher level taxa, the probe intensities were divided by the number of known target phylotypes per probe. The signal intensity above threshold was log10-transformed.

The diversity of the microbial profiles was computed by Simpson’s reciprocal index of diversity (1/D) and Shannon indices on probe-level data [31,32]. Principal component analysis (PCA) and Redundancy analysis (RDA) [33,34] were computed using R packages ‘stats’ and ‘vegan’. The significance of separation in RDA was assessed with a permutation test [33] using 50000 permutations.

Bacterial groups that are associated with the health status were selected with 9-fold cross-validation (CV). The data was split into 9 subsets (folds) of equal size with stratification such that both CD and HC samples were present in all folds. In the CV procedure, eight subsets were used for training, and one subset in turn was left out for testing. Within each CV fold, six random forests were learned to predict the study group using a set of bacteria, selected by: i) choosing the 2, 4, 6, 8, or 10 bacteria having smallest p-values from two-sample *t*-test carried out within the training set, or ii) selecting the bacterial groups with high mean decrease in gini score from random forest using all bacteria [35]. The prediction error of the random forests was then estimated with the test data. The feature selection method with lowest prediction error rate from cross-validation (that is, 8 bacteria with smallest p-values) was then applied for the full data, and its prediction error was estimated with 9-fold cross validation. Significance of the prediction was tested with permutation test using 10,000 permutations.

Differentially present bacterial groups were analyzed with Student’s *t*-test assuming two tailed distribution [36]. False discovery rate (FDR) correction of p-values was carried out using Benjamini-Hochberg (BH) correction [37]. The average relative abundance of each phylum- or genus-like group was estimated by first calculating the percentage of signal intensity within each sample, and then computing the average of percentage of signal intensities within CD and HC groups.

For relative gene expression, the normality of the data was tested by Shapiro-Wilk normality test function. When normality assumption applied, ANOVA and Tukey Honest Significant Differences post-hoc analysis was carried out. If the data residuals differed significantly from normality, Kruskal-Wallis and associated posthoc analysis were carried out [38]. For all analyses, FDR corrected p-values below 0.05 were considered significant. The correlations between bacterial groups, gene expressions and MAMP content were estimated by Spearman coefficient, followed by FDR correction (BH) of p-value.

### Ethical considerations

The study was accepted by the ethical committee of the Hospital District of Southwest Finland. Written informed consent was obtained from all of the study patients or their parents. Pediatric T-CD subjects could not be included as a control group in the study, because in Finland follow-up biopsies are not taken from children if celiac serology turns negative within two years after the implementation of GFD.

## Results

### Composition of the duodenal mucosal microbiota

HITChip microbiota profiles were obtained from 10 CD subjects (4 males and 6 females) and 9 healthy controls (HC, median age 9 ± 4.1 years, 4 males and 5 females) (no significant difference in median age, one HC was not included due to a low reproducibility of HITChip profile, Additional file 2: Table S2).

**Figure 1:**
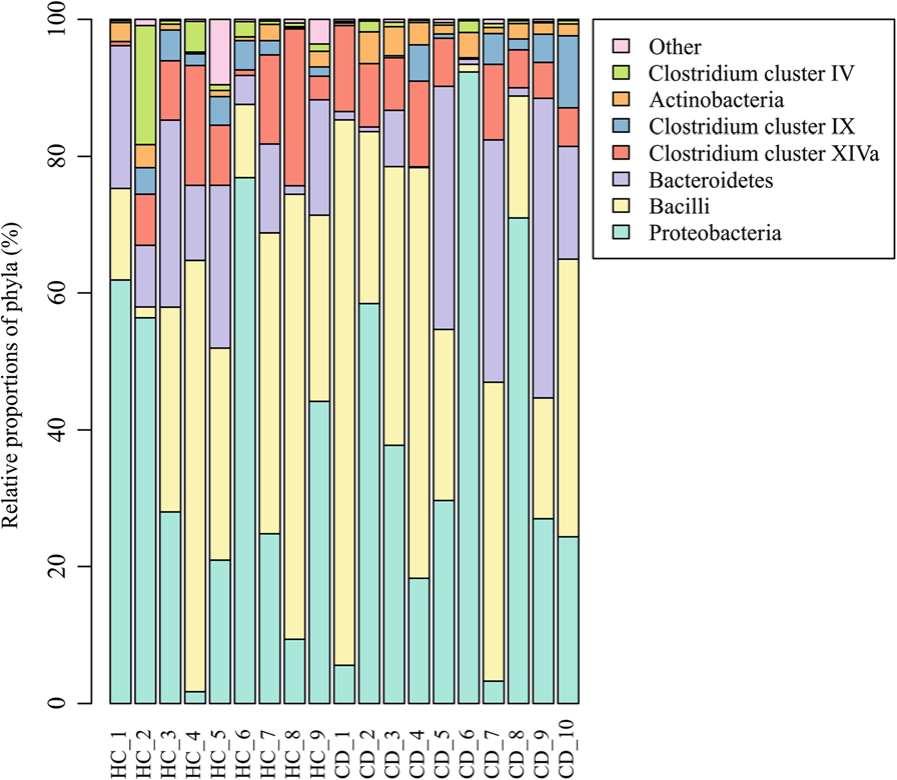
**Composition of the duodenal mucosa-associated microbiota in healthy control (HC and celiac disease (CD) children.** Relative proportions of bacterial phylum-like groups of the total microbiota are depicted for each individual (HC1-HC9 and CD1-CD10).

**Figure 2:**
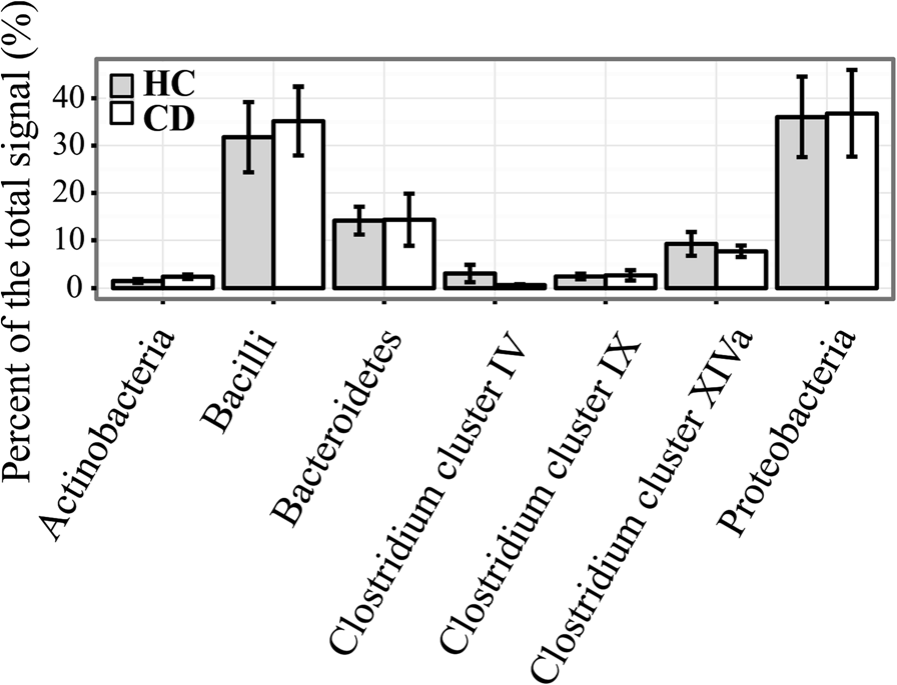
**Relative proportion of phylum-like groups in the duodenal microbiota of celiac disease (CD, white columns) and healthy control children (HC, grey columns).** Mean and standard error are shown for HC (n = 9) and CD (n = 10) groups.

**Figure 3:**
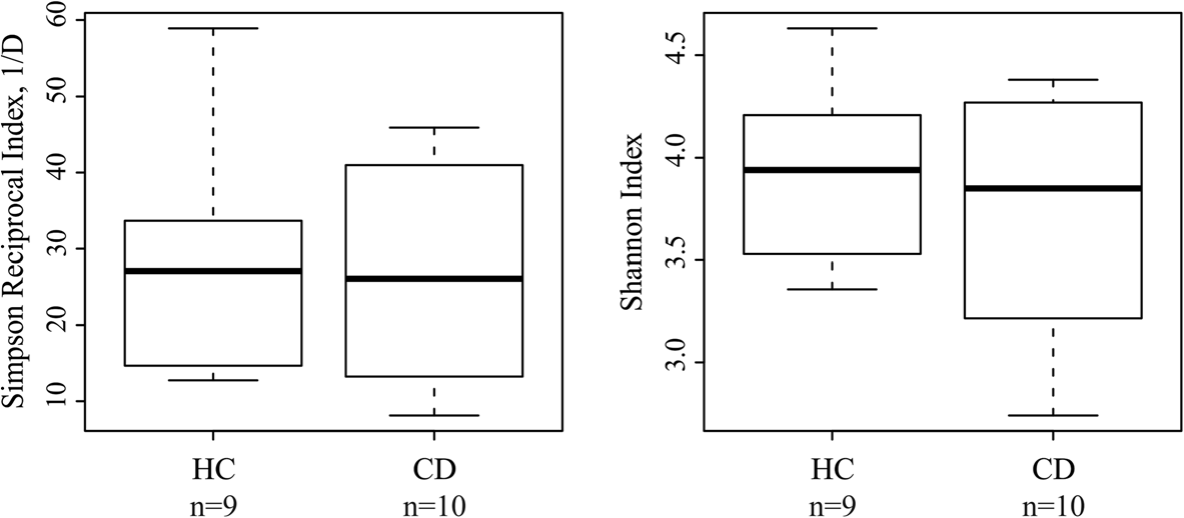
**Duodenal microbiota diversity in healthy control (HC, n = 9) and celiac disease (CD, n = 10) children.** The dominant/common species richness and evenness was assessed by Simpson Reciprocal (1/D) and overall species richness and evenness was assessed by Shannon Indices. Boxplot shows 25th to 75th percentile, with a line at median.

At the bacterial phylum-like level, representatives from 13 groups were detected in the duodenal mucosa of the pediatric subjects (Table 1). The phylum-like level microbiota profiles were found to be individual-specific with large inter-individual variation (Figure 1). Proteobacteria, Bacilli and Bacteroidetes were found to be the major bacterial groups in the duodenal mucosa of both CD and HC (Figure 2). Proteobacteria and Bacilli constituted each approximately one third of the community while Bacteroidetes amounted to around 14% (Figure 2). There were no significant differences in the abundance of bacterial phylum-like groups between CD and HC. Similarly, the bacterial diversity was comparable between the HC and CD groups (Figure 3).

**Table 1:** Composition of the duodenal mucosa-associated microbiota in healthy control (HC) and celiac disease (CD) children: the relative abundance of HITChip genus-like groups and MAMP content estimation

		Relative abundance (%)*				
**Phylum/Order**	**Genus-like phylogenetic group**	**HC**	**CD**	**Gram+/-**	**Flagella**	**GC%**
Actinobacteria	*Propionibacterium*	0.80 ± 0.90	1.43 ± 1.31	G+	N	60
	*Bifidobacterium*	0.52 ± 0.60	0.75 ± 0.98	G+	N	61
Bacilli	*Streptococcus mitis et rel*	13.66 ± 11.64	19.35 ± 12.95	G+	N	40
	*Streptococcus bovis et rel*	9.21 ± 10.79	5.72 ± 3.99	G+	N	37
	*Streptococcus intermedius et rel*	6.38 ± 5.00	7.64 ± 5.12	G+	N	38
	*Gemella*	1.47 ± 2.27	1.77 ± 2.54	G+	N	31
	*Enterococcus*	0.28 ± 0.33	0.19 ± 0.22	G+	Y/N	38
	*Granulicatella*	0.24 ± 0.27	0.23 ± 0.28	G+	N	37
	*Bacillus*	0.17 ± 0.46	0.01 ± 0.01	G+	Y/N	38
	*Aerococcus*	0.16 ± 0.19	0.15 ± 0.19	G+	N	41
Bacteroidetes	*Prevotella melaninogenica et rel*	5.29 ± 4.02	6.51 ± 9.75	G-	N	41
	*Allistipes et rel*	2.03 ± 2.81	2.02 ± 3.37	G-	N	55
	*Parabacteroides distasonis et rel*	1.76 ± 2.34	1.21 ± 2.23	G-	N	45
	*Bacteroides vulgatus et rel*	0.92 ± 1.43	0.47 ± 0.88	G-	N	42
	*Tannerella et rel*	0.88 ± 1.38	0.60 ± 1.08	G-	N	47
	*Bacteroides splachnicus et rel*	0.79 ± 0.69	1.60 ± 2.73	G-	N	43
	*Prevotella tannerae et rel*	0.76 ± 1.10	0.51 ± 0.64	G-	N	47
	*Prevotella oralis et rel*	0.32 ± 0.29	0.16 ± 0.19	G-	N	45
	*Bacteroides intestinalis et rel*	0.32 ± 0.49	0.20 ± 0.29	G-	N	43
	*Prevotella ruminicola et rel*	0.29 ± 0.64	0.11 ± 0.14	G-	N	48
	*Bacteroides plebeius et rel*	0.25 ± 0.30	0.44 ± 0.69	G-	N	44
	*Bacteroides stercoris et rel*	0.17 ± 0.23	0.06 ± 0.07	G-	N	46
	*Bacteroides ovatus et rel*	0.16 ± 0.23	0.08 ± 0.12	G-	N	42
	*Bacteroides fragilis et rel*	0.10 ± 0.13	0.15 ± 0.28	G-	N	44
Clostridium cl. I	*Clostridia*	0.30 ± 0.78	0.01 ± 0.01	G+	Y/N	26
Clostridium cl. III	*Clostridium stercorarium et rel*	0.14 ± 0.23	0.03 ± 0.08	G+	Y	39
Clostridium cl. IV	*Clostridium orbiscindens et rel*	1.14 ± 1.69	0.19 ± 0.17	G+	Y	57
	*Sporobacter termitidis et rel*	0.56 ± 1.31	0.11 ± 0.18	G+	Y	57
	*Clostridium leptum et rel*	0.50 ± 1.07	0.13 ± 0.14	G+	N	50
	*Anaerotruncus colihominis et rel*	0.15 ± 0.41	4E-03 ± 0.01	G+	N	54
	*Ruminococcus callidus et rel*	0.14 ± 0.29	0.06 ± 0.03	G+	N	43
	*Eubacterium siraeum et rel*	0.14 ± 0.40	2E-03 ± 0.01	G+	Y/N	45
	*Ruminococcus bromii et rel*	0.13 ± 0.40	6E-07 ± 2E-06	G+	N	41
	*Papillibacter cinnamivorans et rel*	0.13 ± 0.28	0.01 ± 0.01	G+	N	56
Clostridium cl. IX	*Veillonella*	2.36 ± 1.78	2.61 ± 3.40	G-	N	39
Clostridium cl. XI	*Clostridium difficile et rel*	0.13 ± 0.21	0.06 ± 0.02	G+	Y	29
Clostridium cl. XIVa	*Clostridium symbiosum et rel*	2.79 ± 3.03	3.86 ± 2.51	G+	Y	46
	*Ruminococcus obeum et rel*	1.79 ± 2.28	1.48 ± 0.91	G+	N	42
	*Bryantella formatexigens et rel*	0.90 ± 1.34	0.24 ± 0.11	G+	N	50
	*Coprococcus eutactus et rel*	0.81 ± 1.50	0.21 ± 0.16	G+	N	43
	*Dorea formicigenerans et rel*	0.75 ± 1.07	0.25 ± 0.13	G+	N	41
	*Butyrivibrio crossotus et rel*	0.48 ± 1.12	0.08 ± 0.09	G+	Y	38
	*Eubacterium rectale et rel*	0.47 ± 0.76	0.62 ± 0.53	G+	Y/N	41
	*Ruminococcus gnavus et rel*	0.28 ± 0.51	0.09 ± 0.04	G+	N	43
	*Lachnospira pectinoschiza et rel*	0.26 ± 0.33	0.30 ± 0.18	G+	Y	44
	*Clostridium sphenoides et rel*	0.20 ± 0.44	0.09 ± 0.17	G+	Y	42
	Outgrouping *Clostridium* XIVa	0.20 ± 0.27	0.21 ± 0.18	G+	Y	33
	*Anaerostipes caccae et rel*	0.10 ± 0.17	0.07 ± 0.09	G+	N	44
Clostridium cl. XV	*Eubacterium limosum et rel*	0.21 ± 0.61	4E-03 ± 0.01	G+	N	48
Clostridium cl. XVI	*Solobacterium moorei et rel*	0.52 ± 1.02	0.04 ± 0.11	G+	N	38
Proteobacteria	*Sutterella wadsworthensis et rel*	19.16 ± 13.81	18.78 ± 16.24	G-	N	62
	*Aquabacterium*	9.61 ± 7.08	9.45 ± 9.20	G-	Y	66
	*Xanthomonadaceae*	1.93 ± 2.96	1.90 ± 2.09	G-	Y	61
	*Moraxellaceae*	1.19 ± 1.13	1.33 ± 1.06	G-	N	42
	*Vibrio*	0.86 ± 0.54	1.35 ± 1.11	G-	Y	47
	*Escherichia coli et rel*	0.61 ± 0.65	0.50 ± 0.33	G-	Y	51
	*Enterobacter aerogenes et rel*	0.60 ± 0.90	0.58 ± 0.63	G-	Y	55
	*Burkholderia*	0.45 ± 0.45	0.56 ± 0.47	G-	Y	65
	*Klebisiella pneumoniae et rel*	0.40 ± 0.27	0.74 ± 0.50	G-	N	57
	*Oxalobacter formigenes et rel*	0.29 ± 0.29	0.29 ± 0.33	G-	N	51
	*Haemophilus*	0.25 ± 0.17	0.46 ± 0.31	G-	N	39
	*Pseudomonas*	0.22 ± 0.15	0.40 ± 0.30	G-	Y	65
	*Proteus et rel*	0.17 ± 0.27	0.04 ± 0.04	G-	Y	38
	*Serratia*	0.10 ± 0.07	0.20 ± 0.15	G-	Y	56
Uncult. Mollicutes	Uncultured *Mollicutes*	0.20 ± 0.49	0.03 ± 0.02	G-	N	31
**Summary**	Proportion of Gram+ bacteria	46.1 ± 26.6	45.4 ± 25.5			
	Proportion of Gram- bacteria	52.5 ± 26.7	53.4 ± 25.3			
	Average GC% content of microbiome	48.6 ± 5.8	48.5 ± 6.4			
	Proportion of high-GC% bacteria	32.7 ± 23.3	33.3 ± 27.0			
	Propotion of flagellated bacteria	20.4 ± 8.2	19.9 ± 9.4			

*Relative abundance of the total microbiota (mean ± standard deviation). No statistically significant differences in the abundance of individual genus-like bacterial groups between HC and CD.

*cl* cluster, *uncult* uncultured, *Gram +*  Gram positive bacteria, *Gram-* Gram negative bacteria, *high-GC% bacteria* genomic GC% content is more than 58%, *Y* motile/flagellated, *N* non-motile/non-flagellated, *Y/N* motility variable (50% of the group were taken to be flagellated).

Altogether 65 genus-like groups were detected in the duodenal biopsies (Table 1). HC and CD shared the same predominant genus-like groups, whose average proportion was above 5% of the total signal. The predominant groups were *Sutterella wadsworthensis et rel*., *Streptococcus mitis et rel*., *Aquabacterium*, *Streptococcus bovis et rel*., *Streptococcus intermedius et rel*., and *Prevotella melaninogenica et rel.* (Table 1). Among these, the most abundant bacterial groups in both HC and CD were *Sutterella wadsworthensis et rel*. and *Streptococcus mitis et rel*., both with an average abundance of 14 to 19% (Table 1). No single genus-like bacterial group abundance differed significantly between HC and CD (Table 1).

### The general MAMP content of the duodenal microbiota

The general MAMP content was estimated based on the microbiota profiles (Table 1). No significant difference between the abundance of Gram-positive or Gram-negative bacteria carrying LTA or LPS respectively was found, nor in the abundance of potentially flagellated bacteria between HC and CD. Thus, the ligand load for TLR2, 4 and 5 seems comparable between HC and CD. In addition, the average GC% content of the total microbiota and the relative abundance of high-GC% bacteria (genomic GC% > 58%) were comparable between the groups suggesting a similar load of unmethylated CpG motifs, which are ligands for the TLR9.

### Celiac disease associated microbiota profile

In PCA and RDA plots, HC vs. CD subjects did not cluster separately, showing that the groups do not differ in terms of the total microbiota profile (Additional file 3: Figure S1, Additional file 4: Figure S2). Moreover, as already mentioned above, no single genus-like bacterial group’s abundance differed significantly between HC and CD (Table 1).

**Figure 4:**
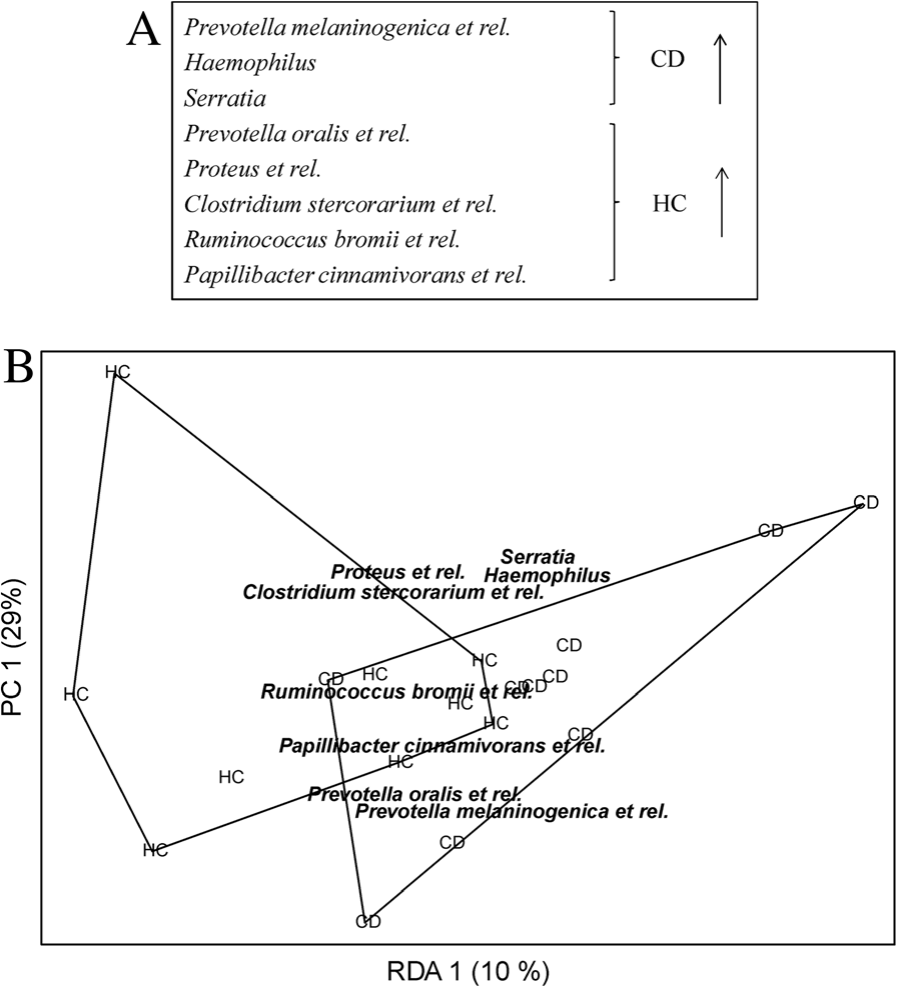
**Sub-profile of the duodenal microbiota separating celiac disease (CD) and healthy control (HC) children. A)** Eight HITChip genus-like level bacterial groups selected with random forest and cross-validation showing higher abundance in either HC (5 groups) or CD (3 groups). The error rate of random forest is 31.6%, whereas 95% confidence intervals for random assignment are [32%, 74%]. **B)** The profile of eight bacterial groups separates healthy control children (HC) and celiac disease children (CD) in redundancy analysis (RDA). p-value obtained by permutation test (50000 permutations) is 0.050.

Further analysis was performed with random forests using feature selection as preprocessing step, where the best method was chosen from six candidates using cross-validation. The aim was to identify a possible health status-related bacterial sub-population within the total microbiota. The feature selection method resulting in lowest cross-validated prediction error was to choose a set of eight genus-like bacteria having lowest p-values from *t*-test (Figure 4). Thus, although no single genus-like group individually differed significantly between HC and CD, a random forest learned with a profile of selected eight genus-like groups predicted the health status with error rate of 31.6%. This is significantly better than random guess, having a median error rate of 53%, and 95% confidence intervals of [32%, 74%].

As can be seen from Table 1, *P. melaninogenica et rel.* has a relative abundance of 5.3% and 6.5% in HC and CD, respectively and it is among the predominant genus-like groups contributing to the separation between HC and CD. Also *Haemophilus* ssp. and *Serratia* ssp. had relatively higher abundance in CD, whereas the other five bacterial groups were higher in HC, see Table 1.

### Human host gene expression

**Figure 5:**
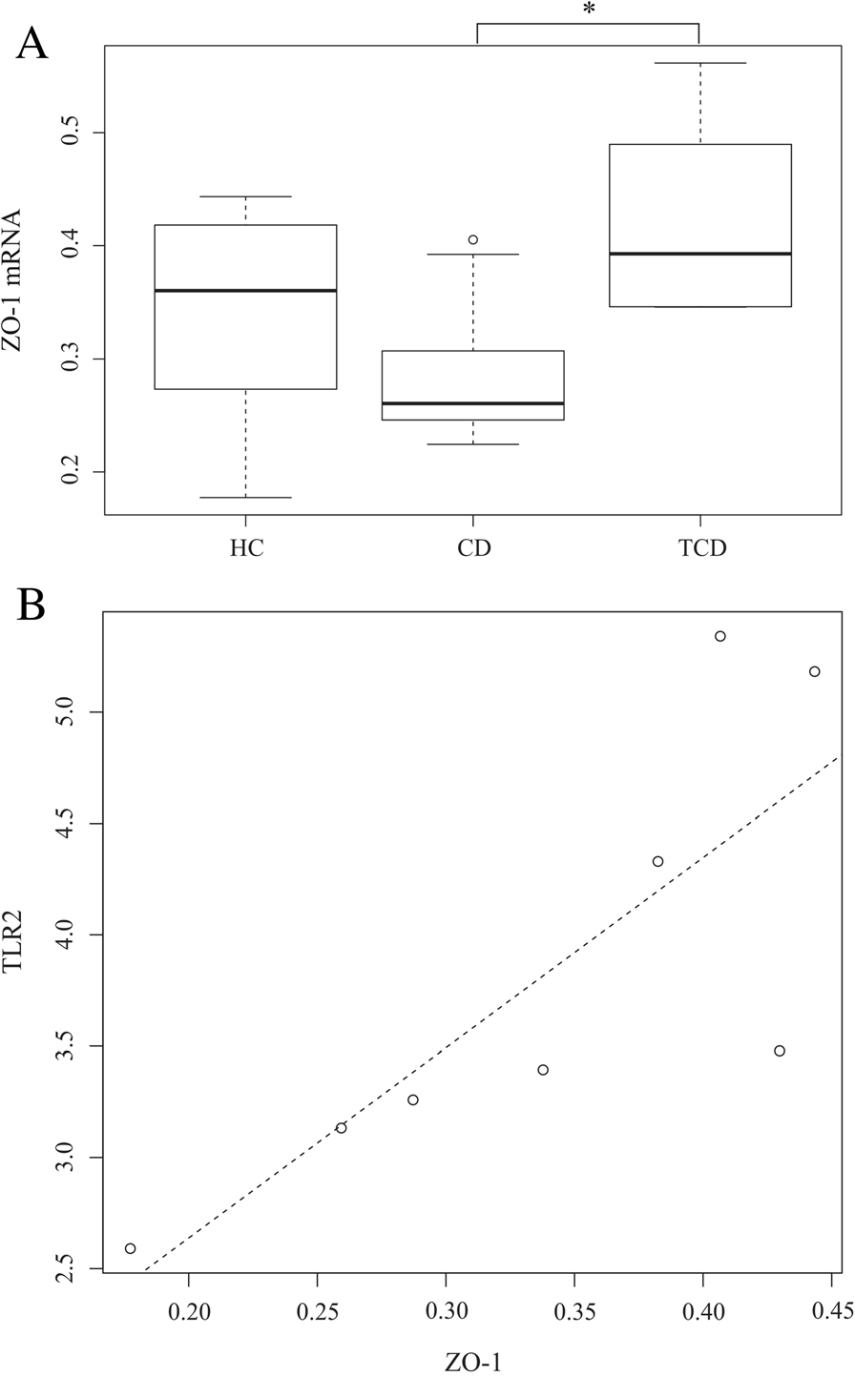
**The relative expression of zonula occludens 1 (ZO-1) (A) and the scatterplot of ZO-1 and TLR2 expressions (B) in duodenal biopsies. A)** Expression of ZO-1 in duodenal biopsies of healthy control children (HC, n = 8), celiac disease children (CD, n = 9) and treated celiac disease adults (T-CD, n = 6). The relative gene expression of ZO-1 is significantly between CD and T-CD (*p < 0.05). Boxplot shows 25th to 75th percentile with a line at median and the whiskers represent interquartile range. Samples, which are outside 1.5 times the interquartile range above the upper quartile (3rd quartile) and below the lower quartile (1st quartile) are denoted by circles. **B)** The scatterplot of relative expression of ZO-1 and TLR2 in duodenal biopsies of HC. The relationship between ZO-1 and TLR2 expressions is estimated by linear regression, and indicated by dashed line. The effect of slope is significant (p = 0.02), having coefficient of determination (R^2^) of 0.63.

**Figure 6:**
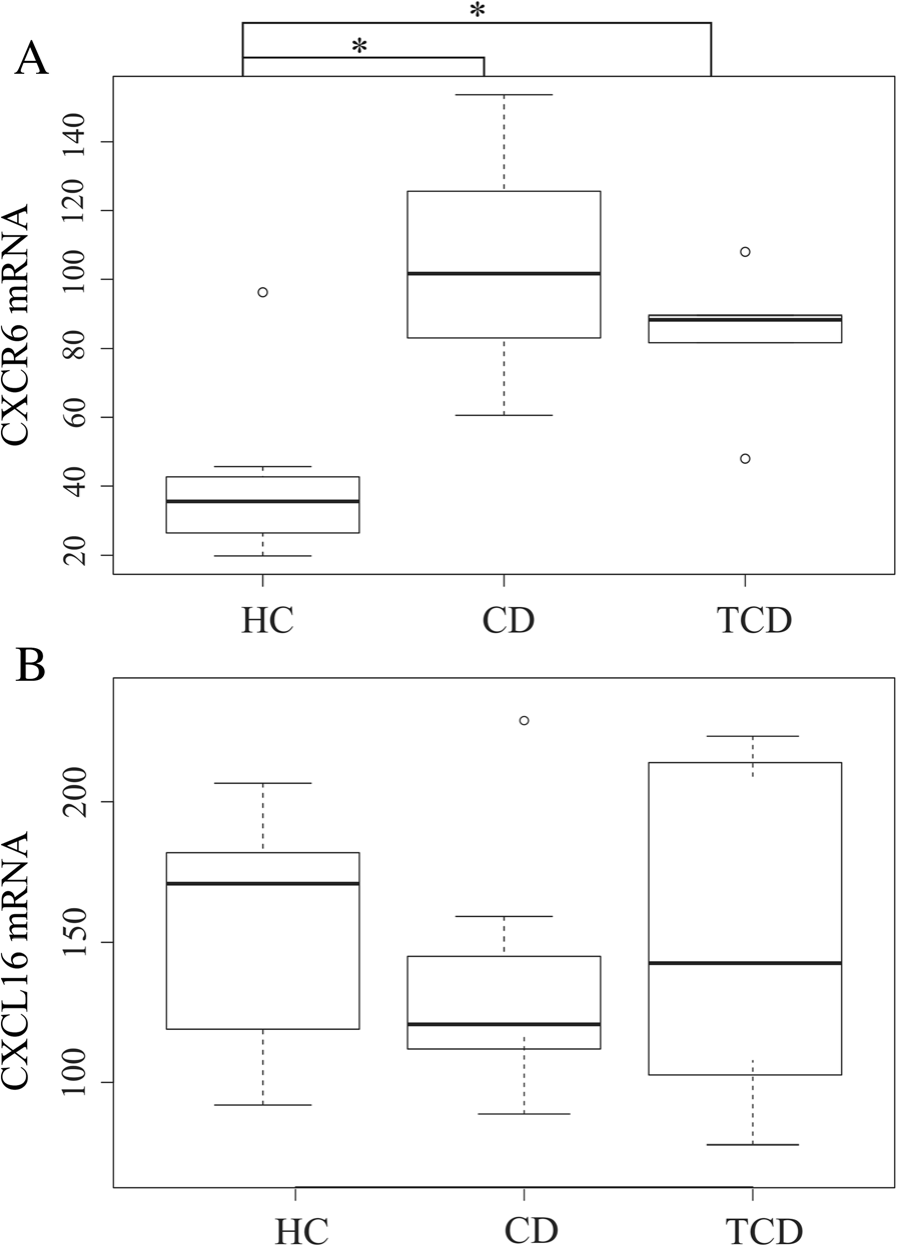
**The relative expression of CXCL6 (A) and CXCR16 (B) in duodenal biopsies of healthy control children (HC, n = 8), celiac disease children (CD, n = 9) and treated celiac adults (T-CD, n = 6).** Boxplot shows 25th to 75th percentile with a line at median and the whisker represents interquartile range. Samples, which are outside 1.5 times the interquartile range above the upper quartile (3rd quartile) and below the lower quartile (1st quartile) are denoted by circles. **A)** The difference in CXCR6 expression between HC and CD or T-CD is statistically significant (*p < 0.05). **B)** CXCL16 expression shows no significant differences between the groups.

The gene expression levels of ZO-1, CXCL16, CXCR6, IL-10, IFN-γ, TNF-α, Cx43, MUC2, and RegIIIγ in duodenal biopsies were assessed by relative quantitative reverse transcription-PCR in the HC and CD children. Biopsies from six T-CD adults were included for comparison, because biopsies from T-CD children were not available (see ethical considerations above). The expression of ZO-1, CXCL16, CXCR6 could be assessed only from 8 HC and 9 CD subjects, because RNA of some of the samples did not suffice after the other qRT-PCR analysis (Figures 5 and 6).

**Figure 7:**
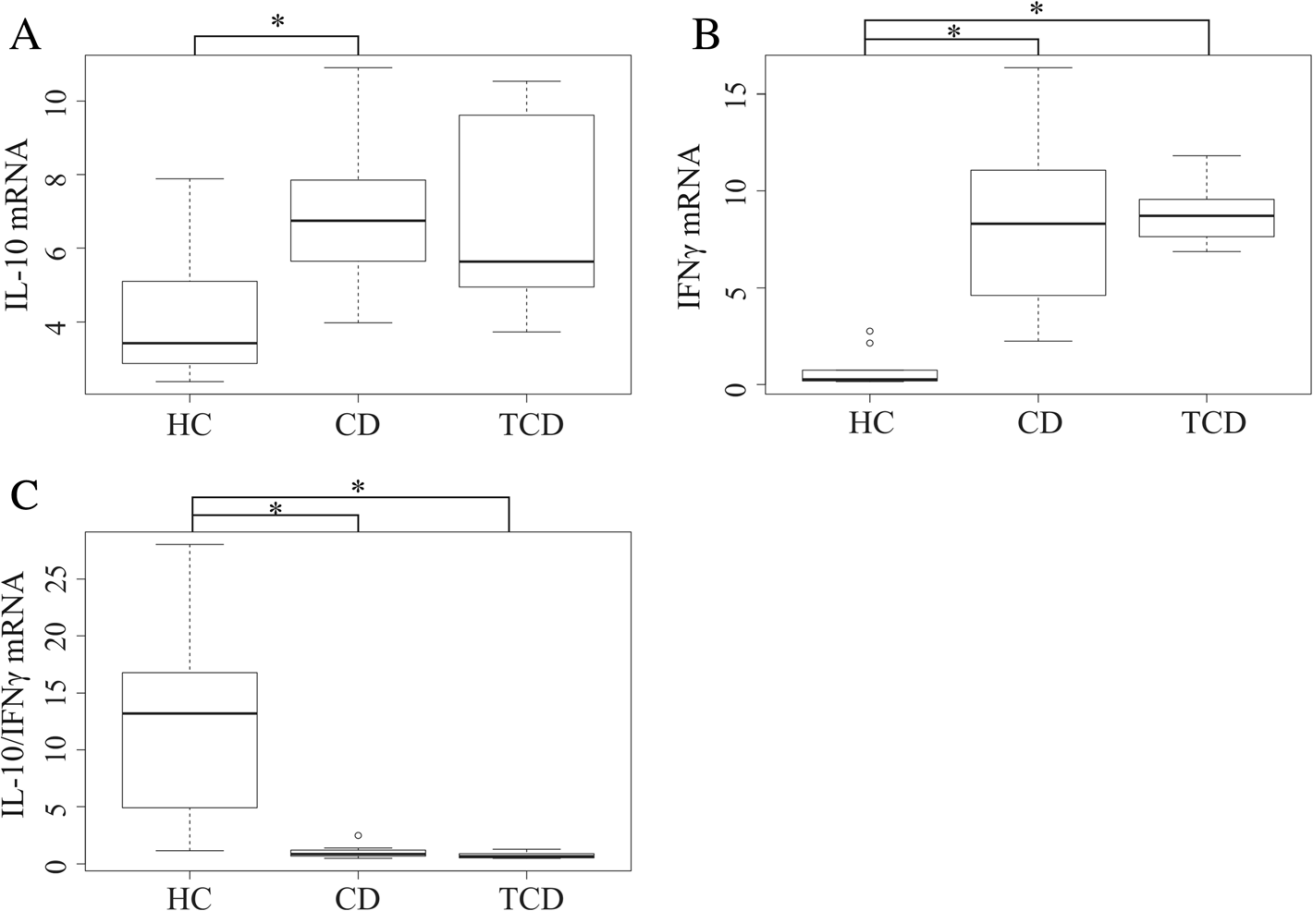
**The relative expression of interleukin-10 (IL-10) (A), interferon gamma (IFN**γ**) (B) and IL-10 to IFN**γ **mRNA ratio (C) in duodenal biopsies of healthy control children (HC, n = 10), celiac disease children (CD, n = 10) and treated celiac adults (T-CD, n = 6).** Boxplot shows 25th to 75th percentile with a line at median and the whisker represents interquartile range. Samples which are outside 1.5 times the interquartile range above the upper quartile (3rd quartile) and below the lower quartile (1st quartile) are denoted by circles. **A**) IL-10 expression is significantly different between HC and CD. **B)** IFNγ expression is significantly different between HC and CD or T-CD. **C)** The ratio of IL-10/IFNγ expression is significantly different between HC and CD or T-CD. The significance between study groups: *p < 0.05.

The gene expression of ZO-1 was decreased in CD as compared to T-CD (Figure 5). The expression of CXCR6 was higher in CD and T-CD as compared to HC (Figure 6A). The expressions of IL-10 and IFN-γ were higher, whereas the ratio of IL-10 to IFN-γ was significantly reduced in both CD and T-CD as compared to HC (Figure 7). The gene expressions of TNF-α, Cx43, MUC2, RegIIIγ (Table A2), and CXCL16 (Figure 6B) were found to be comparable between the groups.

When correlating of TLR2 expression [18] with the expression of proteins related to the physical barrier function or the production of antimicrobial peptides (Cx43, ZO-1, MUC2 and RegIIIγ), the ZO-1 expression correlated positively with the TLR2 expression in the HC group (p = 0.03, rho = 0.88). The expression of Cx43 negatively correlated with the relative abundance of bacteria related to *Solobacterium moorei* (previously *Bulleidia moorei*) *et rel*. (p = 0.03, rho = -0.82). No other correlations were found between the relative abundance of genus-like groups or the estimated MAMP content of the microbiota and the gene expressions (data not shown).

## Discussion

Human small intestine, especially duodenum, is a distinctive environment for microbial life because of the excretion of digestive enzymes and bile, and it harbors a microbiota that is different from the one in the large intestine [39]. The HITChip analysis showed that representatives of the genus *Streptococcus* constitute 29% and 32% of the total duodenal microbiota in HC and CD subjects, respectively, and that the signal obtained for all Bacilli (phylum-like group) predominantly resulted from streptococci. This is in line with the previous findings [39,40]. In addition to Bacilli, Proteobacteria, Bacteroidetes and Clostridium cluster XIVa were found to be the most abundant phylum-like groups in the duodenum. These bacteria have previously been found to be dominant groups in the ileum, distal duodenum and proximal jejunum [17,39,41] and in the duodenum [19]. Interestingly, within Proteobacteria a single most abundant genus-like group was found to be *Sutterella wadsworthensis et rel*. both in CD and HC subjects, which suggests that it belongs to the normal duodenal microbiota. Similarly, Mukhopadhya et al. detected *S. wadsworthensis* with an equally high frequency of 84 to 86% in the colonic biopsies of healthy adults (n = 64) or ulcerative colitis patients (n = 69), showing that the species belongs to the normal microbiota of intestinal mucosa and is unlikely to have a role in IBD [42]. Genus *Aquabacterium* belonging to the Proteobacteria also had a relatively high abundance (~9.5%, prevalence 100% in both CD and HC) in the duodenum of both groups of children. Previously, *Aquabacterium* has been detected in human colonic mucosal biopsy [43], but to our knowledge, this is the first time that *Aquabacterium* has been described as an abundant inhabitant of the human small intestine.

In the PCA and RDA analysis, HC and CD subjects did not cluster separately. Furthermore, none of the 65 genus-like bacterial groups was found to be significantly different in abundance between HC and CD. Thus, the overall duodenal microbiota composition seems comparable between HC and CD, which is in line with the results obtained by Ou et al [17] and Nistal et al [19] using small intestinal biopsies. Moreover, the bacterial diversity was also found to be comparable between the study groups. Previously, bacterial diversity assessed by PCR-T/DGGE has been observed to be increased in children with CD [13,16]. It is noteworthy that PCR-D/TGGE analysis detects only the most abundant bacteria and therefore may strongly underestimate microbiota diversity in complex communities. Our results from high-throughput microbiota profiling, like the results by Nistal et al [19], give a more in depth view of the duodenal microbiota regarding the bacterial groups inhabiting duodenal mucosa and the overall diversity. In addition, several studies have found differences in specific bacterial groups between HC and CD [12,14,15,44]. The most consistent findings from these studies were that children with CD have increased counts of *Bacteroides* and reduced counts of bifidobacteria either in feces or duodenal epithelium [12,14,15] which were not found to differ between CD and HC in this study.

As the overall microbiota profile, diversity or individual genus-like groups did not show a significant difference between HC and CD, random forest was used to explore whether a sub-population bacterial profile could be associated with the health status. A profile of eight bacterial groups was found to distinguish HC from CD. The abundance of *Prevotella melaninogenica,* and the total abundance of *Prevotella spp* (Table 1) were found to be higher in CD, which is in line with the results of Ou et al. [17]*Haemophilus et rel.* were also found to be enriched, although insignificantly as an individual bacterial group, in CD children by Nistal et al. [19]. *Serratia* spp. was also found to be present in higher abundance in CD. The phylogenetic microarray targets mainly *S. marcescens*, which is considered as an opportunistic pathogen able to cause invasive infections (sepsis, meningitis, pneumonitis) in neonates [45,46]. These bacteria may impair the intestinal integrity, but their possible role in CD remains to be elucidated.

In the distinctive profile detected by random forest*, P. oralis, R. bromii*, *P. cinnamivorans*, *Proteus* and *C. stercorarium* groups are increased in the HC group. *R. bromii* is of particular interest, because it acts as key species for fiber/resistant starch degradation in the intestine [47] feeding butyrate producing bacteria including *P. cinnamivorans*[48]. Butyrate is a major source of energy to the enterocytes and acts as a regulator of gene expression, inflammation and differentiation in host cells [49]. Previously, *R. bromii* has been detected at increased level in healthy subjects as compared to Crohn’s disease patients [50], indicating its potential role for benefiting the gut mucosal homeostasis.

Finally, it should be taken into account that the highly individual-specific microbiota-profiles may have a strong impact on the results within small study groups. Moreover, children in the HC group had healthy duodenal mucosa, but had gastrointestinal complaints or other reasons for gastroscopy and the possibility of microbiota alterations in these children as compared to children devoid of any symptoms can´t be excluded. Therefore, the profile comprising eight genus-like bacterial groups, which showed significant difference between CD and HC should be verified in future studies.

Similar average proportion of Gram-positive and Gram-negative bacteria, which are the carriers of LTA and LPS, respectively, was found in CD and HC. Our previous findings from the same cohort showed that the expression of TLR2 is higher in HC [18]. As the LTA content is comparable between HC and CD, the net signaling through TLR2 is presumably higher in HC. *In vitro*, TLR2 stimulation of intestinal epithelial cells (IECs) has been shown to increase Cx43 synthesis, the apical reorganization of ZO-1 and trans-epithelial resistance, which reflects the strength of tight junctions between IECs and barrier function [51-53]. Although no significant difference was found in the expression ZO-1 and Cx43 between HC and CD, the expression of ZO-1 was significantly lower in CD as compared to T-CD. Moreover, there was a significant positive correlation between the expressions of ZO-1 and TLR2 in HC further supporting earlier findings, which demonstrated more permeable epithelial barrier in CD as compared to HC due to the decreased expression of tight junction proteins [54]. Negative correlation was found between the abundance of *S. moorei et rel.* and the expression of Cx43. Previously this bacterium has been associated with oral cavity diseases [55] and its possible down-regulatory effect of Cx43 may facilitate invasion and colonization. In CD, however, *S. moorei et rel.* seems to be irrelevant as it was found to be as abundantly present in HC and CD.

CXCL16 has been shown to function as a scavenger receptor in antigen-presenting cells where it mediates adhesion and phagocytosis of both Gram-positive and Gram-negative bacteria [56]. In addition, it works as a chemokine for CXCR6-expressing cells such as natural killer T cells and T helper 1 (Th1) - polarized CD4 T cells [57]. Since both microbiota alterations and Th1-polarized inflammation have been linked to the pathogenesis of celiac disease, we decided to evaluate the expression of both CXCL16 and its receptor CXCR6 in this study. We found that the expression of cytokine CXCL16 was found to be comparable in CD and HC and T-CD, whereas the expression of its receptor CXCR6 was higher in CD and T-CD as compared to HC. CXCL16 has dual functions as a transmembrane adhesion molecule and a soluble chemokine [58]. Both the membrane-bound form of CXCL16 and its receptor CXCR6 have been found to be expressed not only by dendritic cells/macrophages and T cells respectively, but also by IECs [58-60]. Previously, increased intestinal CXCL16 expression has been observed in the colonic biopsies of Crohn´s disease patients due to immune cell infiltration [60]. In this study, an increased expression of CXCR6 was not only observed in the inflamed mucosa of CD but also in T-CD without immune cell infiltration suggesting an altered expression in epithelial cells. However, increased expression of CXCR6 in mucosa associated immune cells cannot be excluded when studying whole biopsies with different cell types. Diegelmann et al observed that *in vitro* stimulation of CXCR6 activates several distinct signaling pathways in IECs and they suggested that the CXCL16-CXCR6 chemokine-receptor system contributes to the integrity of epithelium and the regulation of mucosal innate and adaptive immune systems [60]. To our knowledge our data report for the first time an increased duodenal expression of CXCR6 in CD subjects in whom it may have an important role in the mucosal immunity.

The average genomic GC content (GC%) of the total microbiota and the proportion of high-GC% bacteria was found to be comparable between the HC and CD groups. High GC content of a bacterial genome correlates with a higher number of potentially immunostimulatory CpG motifs in the genome (Kant R, de Vos WM, Palva A, Satokari R, unpublished results) [29,30] and thus, the GC% was taken to reflect the load of TLR9 ligands in the microbiota. Consequently, HC and CD are likely to harbor similar load of TLR9 ligands. Our previous results from the same cohort showed that the expression of TLR9 in duodenum is higher in CD [18]. Thus, the signaling through TLR9 is presumably higher in CD as compared to HC due to the equally high load of TLR9 ligands and higher expression of TLR9. In immune cells, TLR9 stimulation is known to trigger Th1 type immune responses [61-63]. Further, a recent *in vitro* study demonstrated that apical TLR9 stimulation of IECs leads to an increased expression of IFN-γ and IL-10, but not TNF-α from peripheral blood mononuclear cells (PBMCs) on the basolateral side [64]. In CD patients, increased mucosal expression of IL-10 and IFN-γ is well documented and has been associated with the activity of gliadin-reactive T-cells upon the gluten/gliadin stimulation either *in vivo* or *in vitro*[65-69]. We found that the expression of IL-10 and IFN-γ in the duodenal mucosa was increased not only in CD but also in T-CD with gluten-free diet, suggesting for an additional gluten/gliadin-independent route of stimulation. Similarly to previous studies [65,68,69], TNF-α expression was found to be unaffected in CD and T-CD as compared to HC. The ratio of IL-10 to IFN-γ transcripts was strongly reduced in both CD and T-CD as compared to HC suggesting that although IL-10 expression was also increased there is a significant inclination towards a Th1 response both in untreated and treated CD. Since both CD and T-CD had increased expression of TLR9 [18], we hypothesize that the increased TLR9 signaling in the small intestine may contribute to the persistent activation of Th1 (IFN-γ) signaling pathway markers in the small intestine found in CD children despite gluten-free diet treatment [70]. Due to the limitations in studying whole biopsies with different cell types we cannot conclude which of the proposed signaling routes i.e. the direct TLR9 stimulation of immune cells or the stimulation of IECs with subsequent triggering of immune cells would possibly encompass more into the observed cytokine profile.

## Conclusions

Our results suggest that intestinal microbiota and host-microbe cross-talk play a role in the CD. While the overall microbiota composition in the duodenal mucosa was comparable between the CD and healthy children, a sub-population profile comprising eight genus-like bacterial groups was found to differ significantly between the study groups. The sub-population of bacteria has potentially a specific role in e.g. maintaining gut homeostasis in the healthy individuals or in compromising the epithelial function in CD. Secondly, these and our previous results from the same cohort [18] suggest that altered expression of mucosal receptors and epithelial host-microbe cross-talk have a role in CD. We hypothesize that increased TLR9 signaling in the duodenum may contribute to the Th1 response (increased IFN-γ) found in the small intestinal mucosa of CD subjects even after the implementation of GFD.

## Abbreviations

BH: Benjamini-Hochberg; CD: Celiac disease; CV: Cross validation; Cx43: Connexin-43; CXCR6: Chemokine (C-X-C motif) receptor 6; CXCL16: Chemokine (C-X-C motif) ligand 16; et rel: And relatives; FDR: False discovery rate; GFD: Gluten-free diet; HC: Healthy control; HITChip: Human intestinal tract chip; HLA: Human leukocyte antigen; IECs: Intestinal epithelial cells; IFN-γ: Interferon gamma; IL: Interleukin; LPS: Lipopolysaccharide; LTA: Lipoteichoeic acid; MAMP: Microbe associated molecular pattern; MUC2: Mucin 2; PBMCs: Peripheral blood mononuclear cells; PCA: Principal component analysis; qPCR: Quantitative PCR; RDA: Redundancy analysis; T-CD: Treated celiac disease; TLR: Toll-like receptor; TNF-α: Tumor necrosis factor alpha; ZO-1: Zonula occludens-1.

## Competing interests

The authors declare that they have no competing interest.

## Authors’ contributions

Study concept and design: MK, WdV, RS; collection of clinical samples: MK, HL; acquisition of data: JC, MK, HH, HL, RS; analysis and interpretation of data: JC, MK, HH, JS, RS; statistical analysis: JC, JS; drafting of the manuscript: JC, MK, RS; critical reading and revision of the manuscript: JC, MK, AP, WdV, JS, RS; obtained funding: MK, AP, RS; administrative: MK, AP, RS; study supervision: MK, JS, RS. All authors read and approved the final version for publication.

## Pre-publication history

The pre-publication history for this paper can be accessed here:

http://www.biomedcentral.com/1471-230X/13/113/prepub

## Supplementary Material

Additional file 1: Table S1 Properties of HITChip genus-like groups. Cell wall type (Gram+/Gram-), presence of flagella and the genomic GC% were used for the estimation of microbe associated molecular pattern (MAMP) content in the total microbiota. Click here for file

Additional file 2: Table S2 The duodenal expression levels of nine mucosa-associated genes in celiac disease (CD) and healthy control (HC) children and in treated CD adults (T-CD). Table indicates also from which subjects reproducible HITChip bacterial microarray profiles were obtained. Click here for file

Additional file 3: Figure S1 Principal component analysis (PCA) of the duodenal microbiota profiles from healthy control (HC, n = 9) and celiac disease (CD, n = 10) children at the HITChip genus-like level. The first two principal components capture 35% and 21% of variance, respectively. Click here for file

Additional file 4: Figure S2 Redundancy analysis (RDA) of duodenal microbiota profiles from healthy control (HC, n = 9) and celiac disease (n = 10) children at HITChip genus-like level. The separation is not significant (p = 0.41). Click here for file
