# Supplementary material for: Duodenal microbiota composition and mucosal homeostasis in pediatric celiac disease
Source: BMC Gastroenterol. 2013 Jul 11;13:113. doi: 10.1186/1471-230X-13-113 (PMC3716955; doi:10.1186/1471-230X-13-113)
Supplement: Additional file 1: Table S1 — Properties of HITChip genus-like groups. Cell wall type (Gram+/Gram-), presence of flagella and the genomic GC% were used for the estimation of microbe associated molecular pattern (MAMP) content in the total microbiota. [file 1471-230X-13-113-S1.docx]

**Table S1**. Relative gene expression of nine genes in the duodenal mucosa and indication from which subjects reproducible HITChip bacterial microarray profiles were obtained.

|  | | | |  |  |  |  |  |  |  |  |  |
| --- | --- | --- | --- | --- | --- | --- | --- | --- | --- | --- | --- | --- |
|  |  |  |  |  |  |  |  |  |  |  |  |  |
| **ID** | **Healthy** | **Age** | **IL10** | **TNFα** | **IFNγ** | **CX43** | **ZO1** | **CXCL16** | **CXCR6** | **REG3γ** | **MUC2** | **HITChip** |
|  | **status** | **(Year)** |  |  |  |  |  |  |  |  |  | **profile** |
| HC_10 | HC | 8 | 5.57 | 4.22 | 0.331 | ND | ND | ND | ND | ND | ND | N |
| HC_1 | HC | 7 | 7.89 | 8.45 | 2.14 | ND | ND | ND | ND | ND | ND | Y |
| HC_2 | HC | 16 | 5.11 | 2.71 | 0.182 | 0.168 | 0.443 | 206.61 | 34.05 | 1315.81 | 930555.93 | Y |
| HC_3 | HC | 11 | 3.14 | 3.31 | 0.197 | 0.099 | 0.382 | 168.30 | 45.66 | 1057.08 | 257974.09 | Y |
| HC_4 | HC | 13 | 2.87 | 3.46 | 0.311 | 0.096 | 0.407 | 127.83 | 28.05 | 1591.46 | 585834.38 | Y |
| HC_5 | HC | 6 | 3.64 | 2.09 | 0.742 | 0.082 | 0.287 | 179.73 | 39.69 | 922.69 | 526728.50 | Y |
| HC_6 | HC | 9 | 3.21 | 3.16 | 2.76 | 0.072 | 0.177 | 91.92 | 96.22 | 1354.27 | 111624.95 | Y |
| HC_7 | HC | 5 | 2.86 | 4.96 | 0.184 | 0.115 | 0.430 | 183.89 | 37.01 | 3557.63 | 459498.30 | Y |
| HC_8 | HC | 4 | 2.38 | 2.16 | 0.218 | 0.077 | 0.259 | 110.16 | 19.81 | 29623.75 | 417225.17 | Y |
| HC_9 | HC | 12 | 3.63 | 2.61 | 0.150 | 0.069 | 0.338 | 173.23 | 24.94 | 1278.79 | 434069.54 | Y |
| CD_1 | CD | 11 | 7.66 | 3.58 | 6.30 | ND | ND | ND | ND | ND | ND | Y |
| CD_2 | CD | 3 | 5.64 | 2.47 | 6.53 | 0.184 | 0.232 | 81.43 | 80.59 | 302.44 | 1228528.59 | Y |
| CD_3 | CD | 5 | 10.90 | 4.18 | 16.35 | 0.147 | 0.255 | 159.07 | 153.54 | 428.60 | 1102616.76 | Y |
| CD_4 | CD | 3 | 5.63 | 3.18 | 2.25 | 0.103 | 0.405 | 237.31 | 60.57 | 1194.87 | 1296897.77 | Y |
| CD_5 | CD | 9 | 6.33 | 3.02 | 12.86 | 0.062 | 0.246 | 127.47 | 113.30 | 581.65 | 360840.06 | Y |
| CD_6 | CD | 13 | 7.85 | 3.72 | 11.07 | 0.104 | 0.261 | 111.57 | 128.87 | 1729.09 | 418770.42 | Y |
| CD_7 | CD | 13 | 8.76 | 4.17 | 10.07 | 0.084 | 0.224 | 144.99 | 125.61 | 5300.21 | 426395.69 | Y |
| CD_8 | CD | 7 | 7.02 | 2.91 | 10.15 | 0.131 | 0.262 | 120.73 | 83.09 | 691.99 | 167825.22 | Y |
| CD_9 | CD | 14 | 6.46 | 2.75 | 4.61 | 0.090 | 0.392 | 119.36 | 90.52 | 1081.76 | 609457.74 | Y |
| CD_10 | CD | 10 | 3.98 | 2.10 | 4.38 | 0.086 | 0.307 | 111.94 | 101.65 | 241.78 | 498257.96 | Y |
| TCD_A1 | TCD | 34 | 5.08 | 1.03 | 9.56 | 0.162 | 0.490 | 231.29 | 88.37 | 2531.07 | 698806.42 | NA |
| TCD_A2 | TCD | 53 | 10.53 | 2.76 | 8.16 | 0.133 | 0.561 | 213.97 | 88.36 | 1559.93 | 895458.26 | NA |
| TCD_A3 | TCD | 49 | 6.18 | 0.960 | 6.86 | 0.184 | 0.389 | 154.27 | 89.59 | 1412.79 | 63186.70 | NA |
| TCD_A4 | TCD | 60 | 9.62 | 0.573 | 11.82 | 0.186 | 0.396 | 130.84 | 107.99 | 1668.92 | 225802.20 | NA |
| TCD_A5 | TCD | 30 | 3.73 | 5.15 | 7.63 | 0.084 | 0.346 | 69.32 | 81.69 | 182.93 | 163073.93 | NA |
| TCD_A6 | TCD | 43 | 4.95 | 0.388 | 9.26 | 0.134 | 0.346 | 102.67 | 47.96 | 1273.72 | 209099.97 | NA |

Gene expression levels are normalized against 18S rRNA gene expression

NA: not applicable

HC: healthy controls

CD: celiac disease

TCD: treated celiac disease (gluten free diet > 1 year, negative celiac serology and normal small intestinal mucosa)
