# Supplementary material for: Duodenal microbiota composition and mucosal homeostasis in pediatric celiac disease
Source: BMC Gastroenterol. 2013 Jul 11;13:113. doi: 10.1186/1471-230X-13-113 (PMC3716955; doi:10.1186/1471-230X-13-113)
Supplement: Additional file 2: Table S2 — The duodenal expression levels of nine mucosa-associated genes in celiac disease (CD) and healthy control (HC) children and in treated CD adults (T-CD). Table indicates also from which subjects reproducible HITChip bacterial microarray profiles were obtained. [file 1471-230X-13-113-S2.docx]

**Table S2**. Properties of HITChip genus-like groups. Cell wall type (Gram+/Gram-), presence of flagella and the genomic GC% were used for the estimation of microbial associated molecular pattern (MAMP) content in the total microbiota.

| **Phylum/Order** | **Genus-like phylogenetic group** | **Gram+/Gram-** | **Flagella** | **GC%** | **References** |
| --- | --- | --- | --- | --- | --- |
| Actinobacteria | *Propionibacterium* | G+ | N | 60 | 1,11 |
|  | *Bifidobacterium* | G+ | N | 61 | 1,11 |
| Bacilli | *Streptococcus mitis et rel.* | G+ | N | 40 | 2 |
|  | *Streptococcus bovis et rel.* | G+ | N | 37 | 2,11 |
|  | *Streptococcus intermedius et rel.* | G+ | N | 38 | 2,11 |
|  | *Gemella* | G+ | N | 31 | 2,11 |
|  | *Enterococcus* | G+ | Y/N | 38 | 2,11 |
|  | *Granulicatella* | G+ | N | 37 | 2,11 |
|  | *Bacillus* | G+ | Y/N | 38 | 2,11 |
|  | *Aerococcus* | G+ | N | 41 | 2,11 |
| Bacteroidetes | *Prevotella melaninogenica et rel.* | G- | N | 41 | 3,11 |
|  | *Allistipes et rel.* | G- | N | 55 | 4,11 |
|  | *Parabacteroides distasonis et rel.* | G- | N | 45 | 5,11 |
|  | *Bacteroides vulgatus et rel.* | G- | N | 42 | 1,11 |
|  | *Tannerella et rel.* | G- | N | 47 | 6,11 |
|  | *Bacteroides splachnicus et rel.* | G- | N | 43 | 1,11 |
|  | *Prevotella tannerae et rel.* | G- | N | 47 | 3,11 |
|  | *Prevotella oralis et rel.* | G- | N | 45 | 3,11 |
|  | *Bacteroides intestinalis et rel.* | G- | N | 43 | 1,11 |
|  | *Prevotella ruminicola et rel.* | G- | N | 48 | 3,11 |
|  | *Bacteroides plebeius et rel.* | G- | N | 44 | 1,11 |
|  | *Bacteroides stercoris et rel.* | G- | N | 46 | 1,11 |
|  | *Bacteroides ovatus et rel.* | G- | N | 42 | 1,11 |
|  | *Bacteroides fragilis et rel.* | G- | N | 44 | 1,11 |
| Clostridium cl. I | *Clostridia* | G+ | Y/N | 26 | 1,2 |
| Clostridium cl. III | *Clostridium stercorarium et rel.* | G+ | Y | 39 | 2 |
| Clostridium cl. IV | *Clostridium orbiscindens et rel.* | G+ | Y | 57 | 2 |
|  | *Sporobacter termitidis et rel.* | G+ | Y | 57 | 2 |
|  | *Clostridium leptum et rel.* | G+ | N | 50 | 2,11 |
|  | *Anaerotruncus colihominis et rel.* | G+ | N | 54 | 2,11 |
|  | *Ruminococcus callidus et rel.* | G+ | N | 43 | 2 |
|  | *Eubacterium siraeum et rel.* | G+ | Y/N | 45 | 2,11 |
|  | *Ruminococcus bromii et rel.* | G+ | N | 41 | 2,11 |
|  | *Papillibacter cinnamivorans et rel.* | G+ | N | 56 | 2 |
| Clostridium cl. IX | *Veillonella* | G- | N | 39 | 1,11 |
| Clostridium cl. XI | *Clostridium difficile et rel.* | G+ | Y | 29 | 2,11 |
| Clostridium cl. XIVa | *Clostridium symbiosum et rel.* | G+ | Y | 46 | 2 |
|  | *Ruminococcus obeum et rel.* | G+ | N | 42 | 2 |
|  | *Bryantella formatexigens et rel.* | G+ | N | 50 | 2 |
|  | *Coprococcus eutactus et rel.* | G+ | N | 43 | 2,11 |
|  | *Dorea formicigenerans et rel.* | G+ | N | 41 | 2,11 |
|  | *Butyrivibrio crossotus et rel.* | G+ | Y | 38 | 1,2,11 |
|  | Eubacterium rectale et rel. | G+ | Y/N | 41 | 2,11 |
|  | Ruminococcus gnavus et rel. | G+ | N | 43 | 2 |
|  | Lachnospira pectinoschiza et rel. | G+ | Y | 44 | 2 |
|  | Clostridium sphenoides et rel. | G+ | Y | 42 | 2 |
|  | Outgrouping clostridium cl. XIVa | G+ | Y | 33 | 2 |
|  | *Anaerostipes caccae et rel.* | G+ | N | 44 | 2,11 |
| Clostridium cl. XV | *Eubacterium limosum et rel.* | G+ | N | 48 | 2,11 |
| Clostridium cl. XVI | *Solobacterium moorei et rel.* | G+ | N | 38 | 2 |
| Proteobacteria | *Sutterella wadsworthensis et rel.* | G- | N | 62 | 7,11 |
|  | *Aquabacterium* | G- | Y | 66 | 8 |
|  | *Xanthomonadaceae* | G- | Y | 61 | 9,11 |
|  | *Moraxellaceae* | G- | N | 42 | 1,11 |
|  | *Vibrio* | G- | Y | 47 | 9,11 |
|  | *Escherichia coli et rel.* | G- | Y | 51 | 1,9,11 |
|  | *Enterobacter aerogenes et rel.* | G- | Y | 55 | 9,11 |
|  | *Burkholderia* | G- | Y | 65 | 9,11 |
|  | *Klebisiella pneumoniae et rel.* | G- | N | 57 | 9 |
|  | *Oxalobacter formigenes et rel.* | G- | N | 51 | 9,11 |
|  | *Haemophilus* | G- | N | 39 | 9,11 |
|  | *Pseudomonas* | G- | Y | 65 | 9,11 |
|  | *Proteus et rel.* | G- | Y | 38 | 9,11 |
|  | *Serratia* | G- | Y | 56 | 9,11 |
| Uncultured Mollicutes | Uncult. Mollicutes | G- | N | 31 | 10,11 |

cl. – cluster; uncult. – uncultured; Gram+ – Gram positive bacteria; Gram- – Gram negative bacteria; GC% – genomic GC content (%); Y – motile/flagellated; N – non-motile/non-flagellated, Y/N – motility variable

**References**

1. Dworkin M, Falkow S, Rosenberg E, Schledfer KH, Stackebrandt E (Eds): **The Prokaryotes: a handbook on the biology of bacteria**. 3nd edition. New York: Springer; 2006.

2. de Vos P, Garrity GM, Jones D, Krieg NR, Ludwig W, Rainey FA, Schleifer KH, Whitman WB (Eds): **The Firmicutes.** In *Bergey's manual of systematic bacteriology. Volume 3.* 2nd edition. New York: Springer; 2009.

3. Shah HN, Collins DM: ***Prevotella*, a new genus to include *Bacteroides melaninogenicus* and related species formerly classified in the genus *Bacteroides***. *Int J Syst Bacteriol* 1990, **40**: 205-208.

4. Rautio M, Eerola E, Väisänen-Tunkelrott ML, Molitoris D, Lawson P, Collins MD, Jousimies-Somer H: **Reclassification of *Bacteroides putredinis* (Weinberg et al., 1937) in a new genus *Alistipes* gen.nov., as *Alistipes putredinis* comb. nov., and description of *Alistipes finegoldii* sp. nov., from human sources.** *System Appl Microbiol* 2003, **26:** 182-188.

5. Sakamoto M, Benno Y: **Reclassification of *Bacteroides distasonis, Bacteroides goldsteinii* and *Bacteroides medae* as *Parabacteroides distasonis* gen. nov., comb. nov., *Parabacteroides goldsteinii* comb. nov. and *Parabacteroides merdae* comb. nov.** *Int J Syst Evol Microbiol* 2006**, 56**: 1599-1605.

6. Sakamoto M, Suzuki M, Umeda M, Ishikawa I, Benno Y: **Reclassification of *Bacteroides forsythus* (Tanner et al.1986) as *Tannerella forsythensis* corrig., gen. nov., comb. nov.** *Int J Syst Evol Microbiol* 2002, **52:** 841-849.

7. Wexler HM, Reeves D, Summanen PH, Molitoris E, McTeague M, Duncan J, Wilson KH, Finegold SM: ***Sutterella wadsworthensis* gen. nov., sp. nov., bile-resistant microaerophilic *Campylobacter gracilis*-like clinical isolates**. *Int J Syst Bacteriol* 1996, **46:** 252-258.

8. Kalmbach S, Manz W, Wecke J, Szewzyk U. ***Aquabacteium* gen. nov., with description of *Aquabacterium citratiphilum* sp. nov., *Aquabacterium parvum* sp.nov. and *Aquabacterium commune* sp. nov., three *in situ* dominant bacterial species from the Berlin drinking water system.** *Int J Syst Bacteriol* 1999, **49:** 769-777.

9. Garrity GM, Brenner DJ, Krieg NR, Staley JT (Eds) **The Proteobacteria***.* In *Bergey's manual of systematic bacteriology. Volume 2.* 2nd edition. New York: Springer; 2005.

10. Wolgenuth CW, Igoshin O, Oster G: **The motility of *Mollicutes*.** *Biophys J* 2003, **85:** 828-842.

11. The National Center for Biotechnology Information database for Microbial Genomes [http://www.ncbi.nlm.nih.gov/genome] (date of access: 19 October 2012).
