# Supplementary figures and images for: Duodenal microbiota composition and mucosal homeostasis in pediatric celiac disease
Source: BMC Gastroenterol. 2013 Jul 11;13:113. doi: 10.1186/1471-230X-13-113 (PMC3716955; doi:10.1186/1471-230X-13-113)

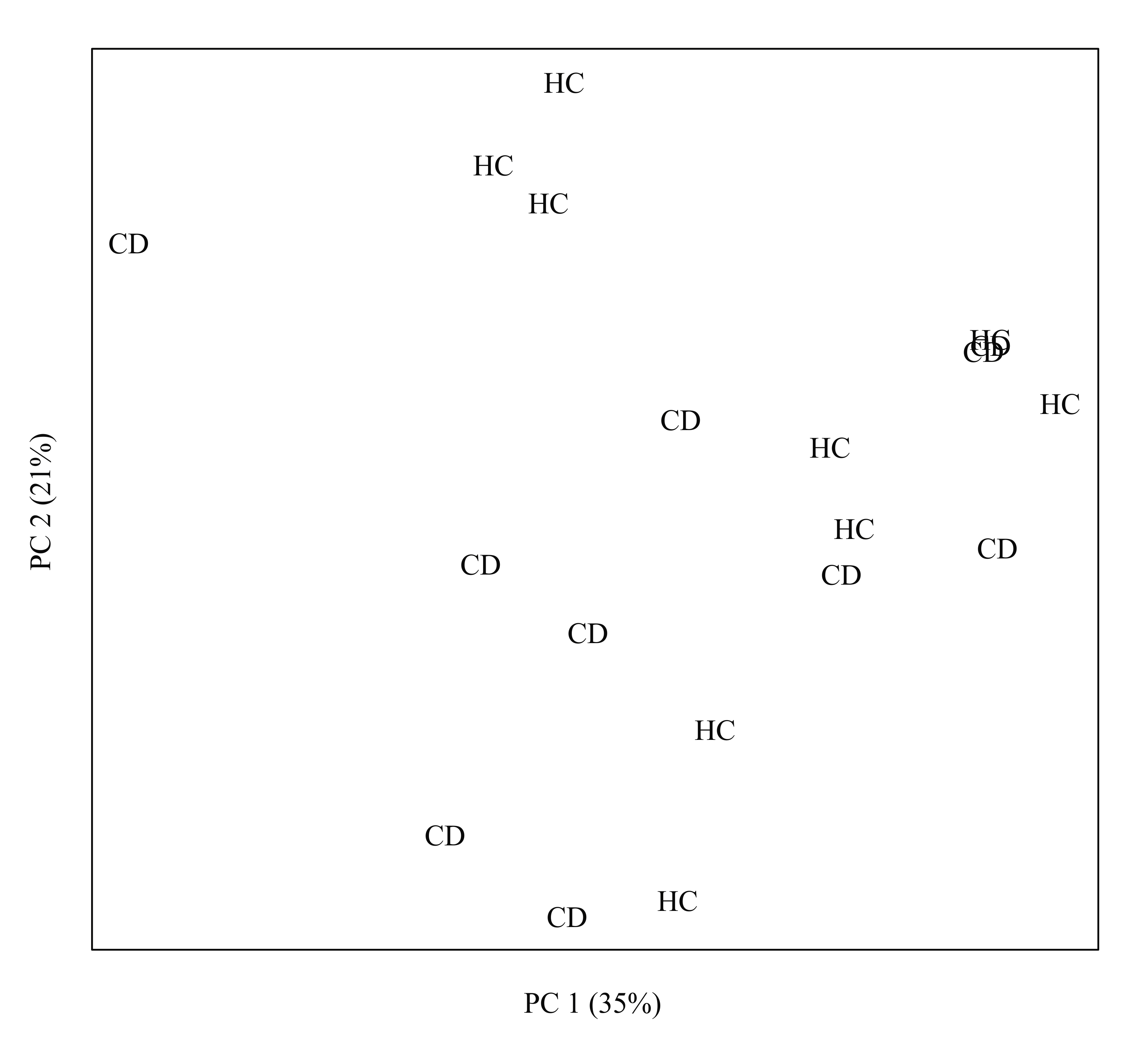

Supplement: Additional file 3: Figure S1 — Principal component analysis (PCA) of the duodenal microbiota profiles from healthy control (HC, n = 9) and celiac disease (CD, n = 10) children at the HITChip genus-like level. The first two principal components capture 35% and 21% of variance, respectively. [file 1471-230X-13-113-S3.tiff]

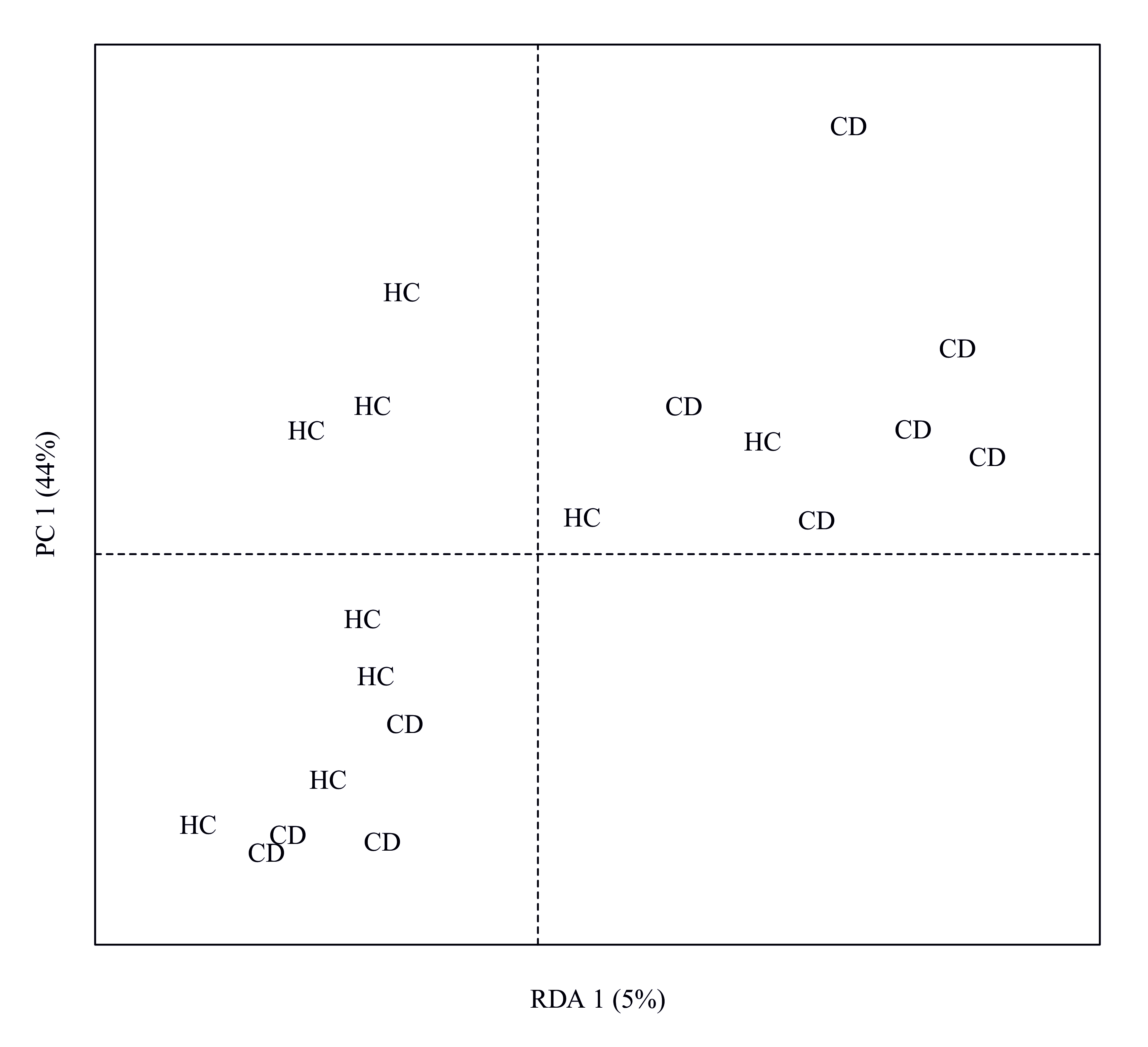

Supplement: Additional file 4: Figure S2 — Redundancy analysis (RDA) of duodenal microbiota profiles from healthy control (HC, n = 9) and celiac disease (n = 10) children at HITChip genus-like level. The separation is not significant (p = 0.41). [file 1471-230X-13-113-S4.tiff]
